# Supplementary material for: Preference for and resistance to a toxic sulfur volatile opens up a unique niche in Drosophila busckii
Source: Nat Commun. 2025 Jan 17;16:767. doi: 10.1038/s41467-025-55971-2 (PMC11742422; doi:10.1038/s41467-025-55971-2)
Supplement: Supplementary file 1 — Supplementary Information [file 41467_2025_55971_MOESM1_ESM.pdf]

Supplementary materials:

Supplementary figures:

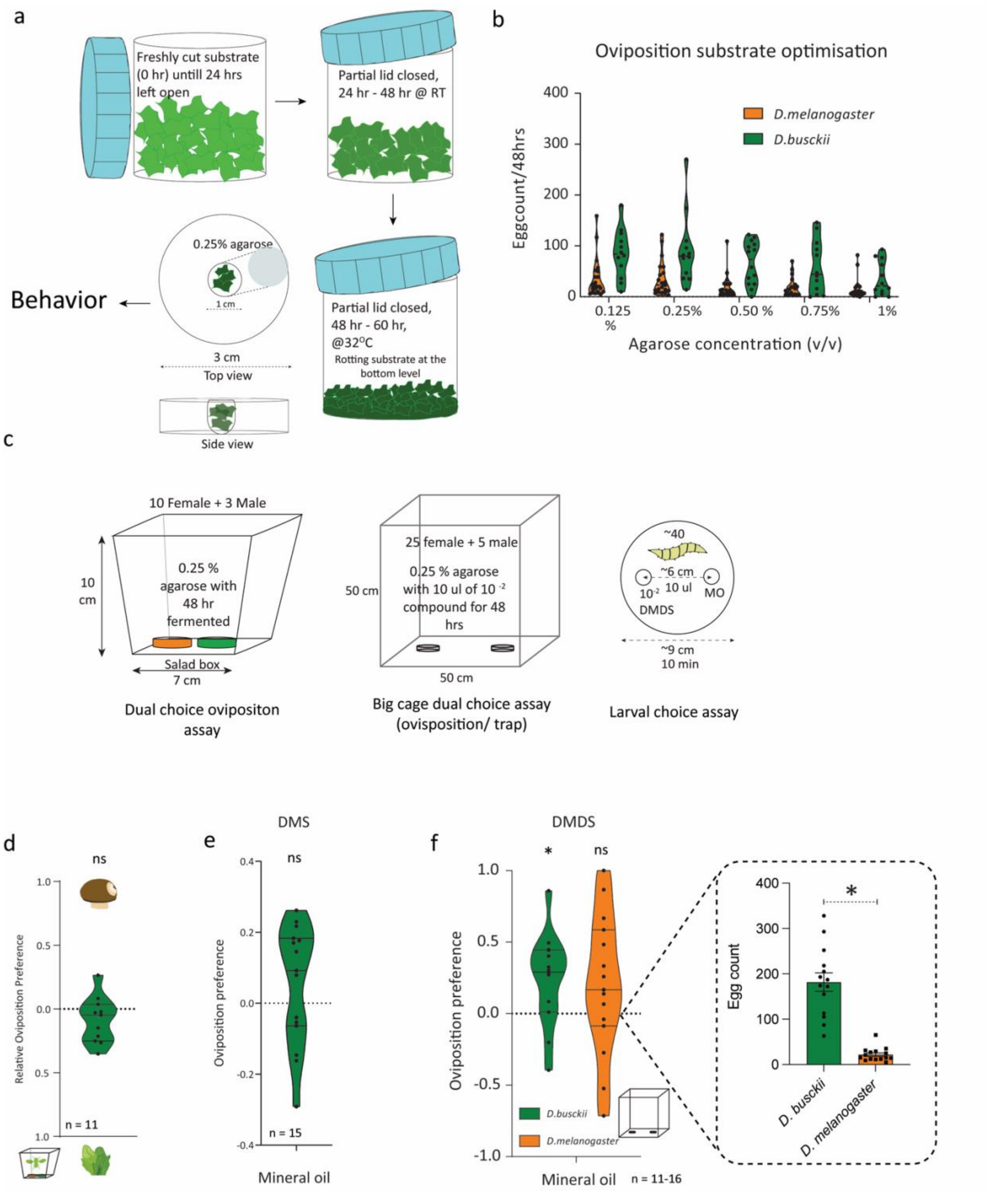

Supplementary fig 1: **Methodological details and behavioral tests related to figure 1.**

a. A schematic representation of artificial rotting protocol is explained earlier in methods.

b. Concentration optimization of agarose plates for experiments testing egg-laying behaviour in *Dmel* and *Dbus*. 0.25% agarose was selected as the final concentration for all the experiments in the study.
c. Schematic representation of bioassays used in subsequent experiments.
d. Binary choice assay testing oviposition preference between rotting mushrooms and rotting spinach. Significance was tested using a two tailed, unpaired t-test with Welch's correction. *: p = 0.384. n = 11
e. Binary choice assay testing oviposition preference between DMS and mineral oil for *D. busckii*. Significance was tested using a two tailed, unpaired t-test with Welch's correction. *: p = 0.241. n = 14
f. Binary choice assay testing oviposition preference between DMDS and mineral oil in *D. busckii* and *D. melanogaster*. The egg count per species is shown in the figure inset. Significance was tested using a two tailed, unpaired t-test with Welch's correction. *: $p < 0.05$. n = 11-16. Error bars represent mean $\pm$ SEM. Source data are provided as a source data file.

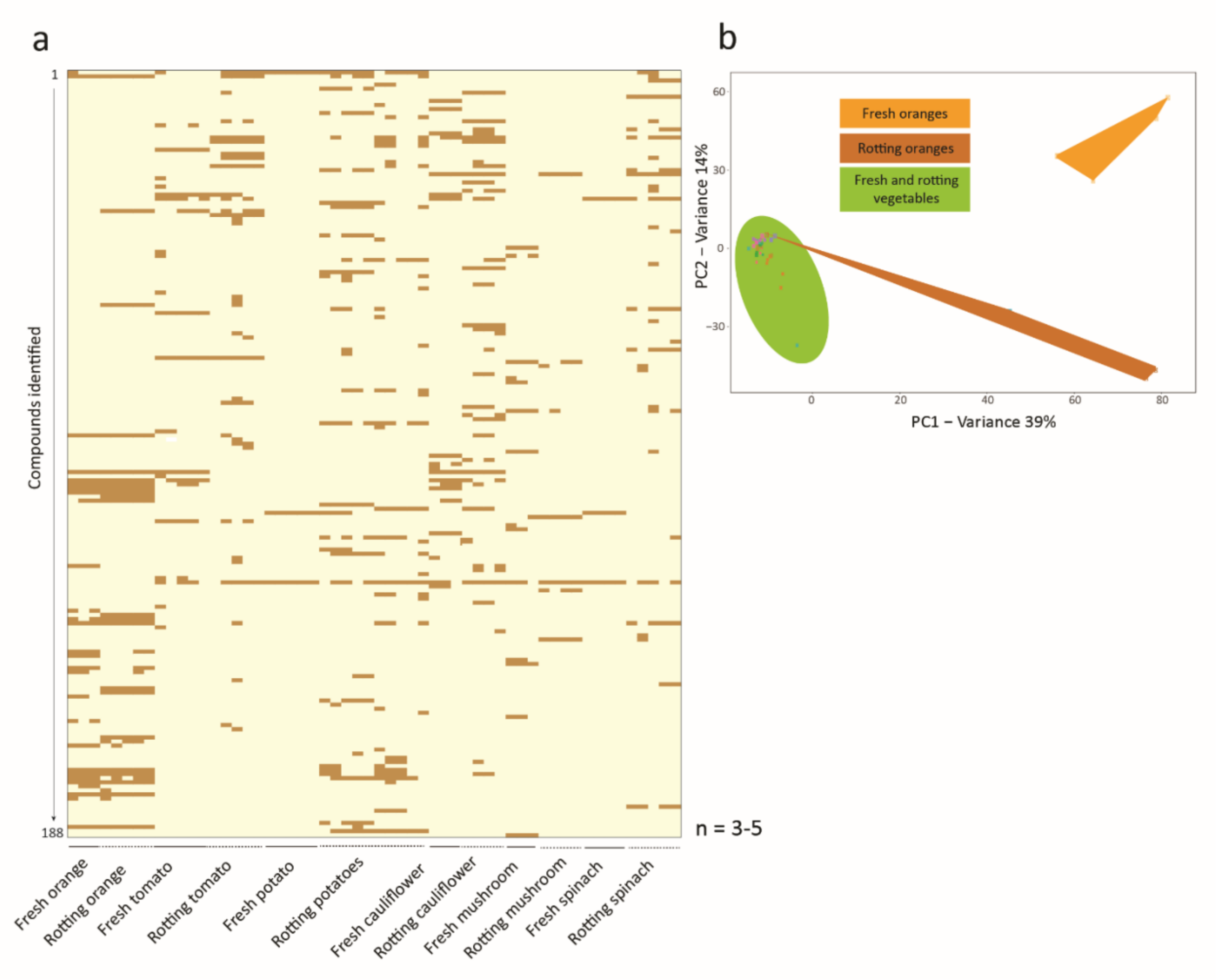

Supplementary fig 2: **Chemical analysis of substrates in two stages**

a. Heatmap showing a total of 188 chemical compounds identified from five ecologically relevant substrates in *D. busckii* when tested in both fresh and rotting stages. The x-axis depicts alternate bold and dashed lines where each alternating segment represents multiple replicates from the same category of stimulus depicted below.
b. A principal component analysis of all stimuli chromatograms generated using XCMS software[74] shows a clear distinction between fresh and rotting oranges from another group (collectively termed vegetables here)

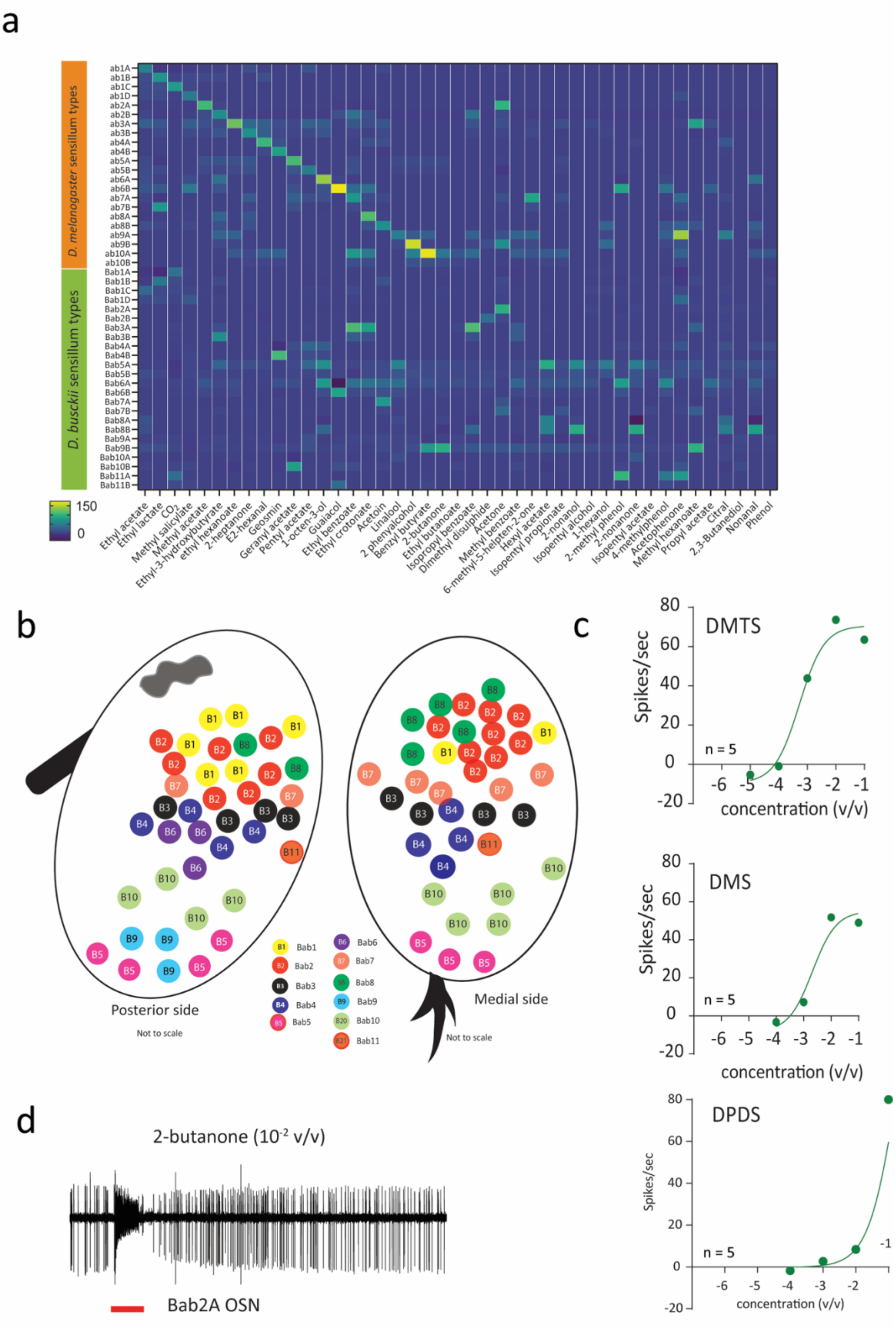

a
D. melanogaster sensillum types
D. busckii sensillum types
ab1A
ab1B
ab1C
ab1D
ab2A
ab2B
ab3A
ab3B
ab4A
ab4B
ab5A
ab5B
ab6A
ab6B
ab7A
ab7B
ab8A
ab8B
ab9A
ab9B
ab10A
ab10B
Bab1A
Bab1B
Bab1C
Bab1D
Bab2A
Bab2B
Bab3A
Bab3B
Bab4A
Bab4B
Bab5A
Bab5B
Bab6A
Bab6B
Bab7A
Bab7B
Bab8A
Bab8B
Bab9A
Bab9B
Bab10A
Bab10B
Bab11A
Bab11B
150
0
Ethyl acetate
Ethyl lactate
CO2
Methyl salicylate
Methyl acetate
Ethyl-3-hydroxybutyrate
ethyl hexanoate
2-heptanone
E2-hexanal
Geosmin
Geranyl acetate
Pentyl acetate
1-octen-3-ol
Guaiacol
Ethyl benzoate
Ethyl crotonate
Acetoin
Linalool
2 phenylalcohol
Benzyl butyrate
2-butanone
Ethyl butanoate
Isopropyl benzoate
Dimethyl disulphide
Acetone
Methyl benzoate
6-methyl-5-helpten-2-one
Hexyl acetate
Isopentyl propionate
2-nonanol
Isopentyl alcohol
1-hexanol
2-methyl phenol
2-nonanone
Isopentyl acetate
4-methylphenol
Acetophenone
Methyl hexanoate
Propyl acetate
Citral
2,3-Butanediol
Nonanal
Phenol
b
Posterior side
Not to scale
Bab1
Bab2
Bab3
Bab4
Bab5
Bab6
Bab7
Bab8
Bab9
Bab10
Bab11
Medial side
Not to scale
c
DMTS
DMS
DPDS
Spikes/sec
concentration (v/v)
n = 5
d
2-butanone (10-2 v/v)
Bab2A OSN

Supplementary fig 3: **Classification of basiconic type sensilla on the antenna of *D. busckii***

a. Heatmap of antennal OSNs innervating ten established basiconic types in *Dmel* and eleven basiconic types identified from this study in *Dbus* with a panel of 43 ecologically relevant odors (see methods and supplementary table 1 for a list of odorants). Acetone was freshly pipetted during each odor delivery and therefore, slight, unspecific, activation of the Bab2B neuron (otherwise responding only to DMDS) can be observed in the heatmap. n = 3 for *Dmel* while n = 2-8 for *Dbus.* Some sensillum types (Bab9) were extremely rare to encounter and hence have a low replicate value (n=2).
b. A spatial distribution map of all sensillum classes identified in *Dbus* using a panel of 43 diverse odorants.
c. The dose-response properties of the Bab2B OSNs when tested against multiple short-chain oligosulfides. n = 5. Each replicate corresponds to a single Bab2B OSN recording from an individual fly.
d. A representative trace of Bab2A OSN type when excited by freshly pipetted 2-butanone (10^{-2} v/v in mineral oil). Source data are provided as a source data file.

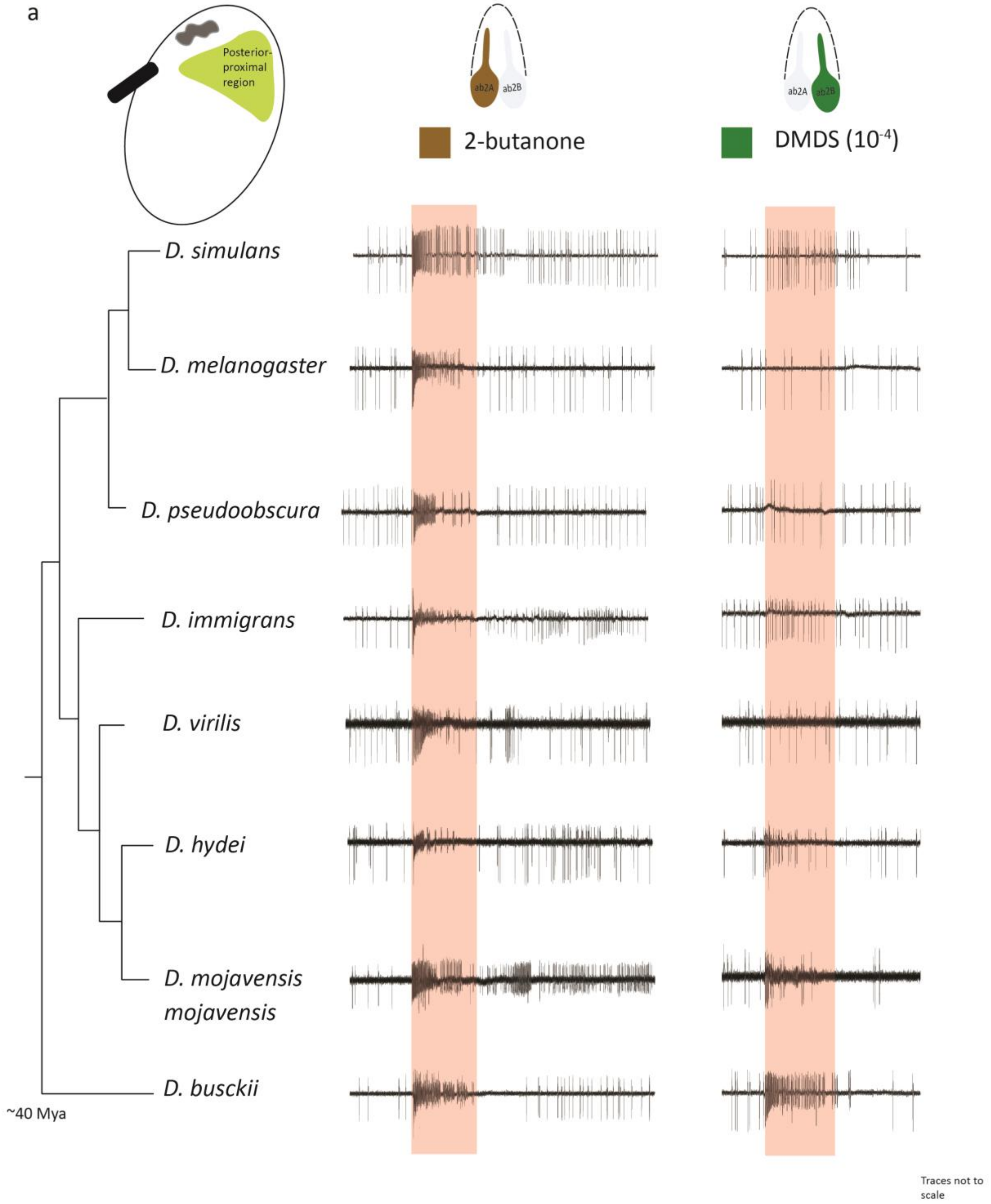

a
Posterior-proximal region
ab2A
ab2B
2-butanone
DMDS (10^{-4})
D. simulans
D. melanogaster
D. pseudoobscura
D. immigrans
D. virilis
D. hydei
D. mojavensis mojavensis
D. busckii
~40 Mya
Traces not to scale

Supplementary fig 4: **Traces of OSNs responding to test odors across species.**

a. Sample traces of OSNs responding to 2-butanone and DMDS when tested across multiple drosophilid species. Phylogenetic branch lengths are representative and not to scale.

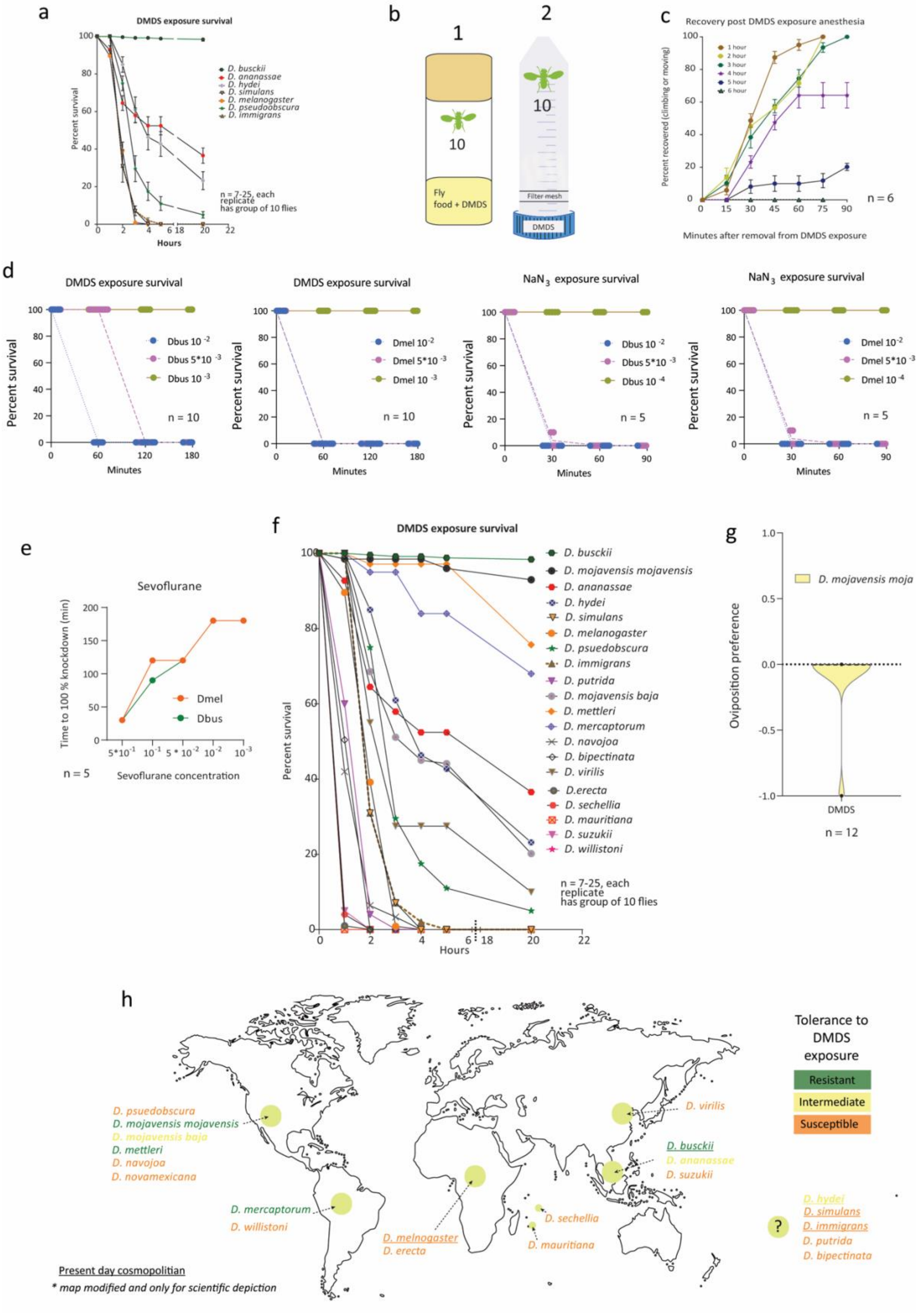

a
DMDS exposure survival
Percent survival
Hours
D. busckii
D. ananassae
D. hydei
D. simulans
D. melanogaster
D. pseudoobscura
D. immigrans
n = 7-25, each replicate has group of 10 flies
b
1
2
10
Fly food + DMDS
Filter mesh
DMDS
c
Recovery post DMDS exposure anesthesia
Percent recovered (climbing or moving)
Minutes after removal from DMDS exposure
1 hour
2 hour
3 hour
4 hour
5 hour
6 hour
n = 6
d
DMDS exposure survival
Percent survival
Minutes
Dbus 10 -2
Dbus 5*10 -3
Dbus 10 -3
n = 10
DMDS exposure survival
Dmel 10-2
Dmel 5*10 -3
Dmel 10 -3
n = 10
NaN3 exposure survival
Dbus 10 -2
Dbus 5*10 -3
Dbus 10 -4
n = 5
NaN3 exposure survival
Dmel 10-2
Dmel 5*10 -3
Dmel 10 -4
n = 5
e
Sevoflurane
Time to 100 % knockdown (min)
Dmel
Dbus
5*10-1
10-1
5 * 10-2
10-2
10-3
n = 5
Sevoflurane concentration
f
DMDS exposure survival
Percent survival
Hours
D. busckii
D. mojavensis mojavensis
D. ananassae
D. hydei
D. simulans
D. melanogaster
D. psuedobscura
D. immigrans
D. putrida
D. mojavensis baja
D. mettleri
D. mercaptorum
D. navojoa
D. bipectinata
D. virilis
D.erecta
D. sechellia
D. mauritiana
D. suzukii
D. willistoni
n = 7-25, each replicate has group of 10 flies
g
Oviposition preference
D. mojavensis moja
DMDS
n = 12
h
Tolerance to DMDS exposure
Resistant
Intermediate
Susceptible
D. psuedobscura
D. mojavensis mojavensis
D. mojavensis baja
D. mettleri
D. navojoa
D. novamexicana
D. virilis
D. busckii
D. ananassae
D. suzukii
D. mercaptorum
D. willistoni
D. melnogaster
D. erecta
D. sechellia
D. mauritiana
D. hydei
D. simulans
D. immigrans
D. putrida
D. bipectinata
Present day cosmopolitian
* map modified and only for scientific depiction

Supplementary fig 5: **Supplementary information related to the main figure 3.**

a. A time course representation of DMDS induced susceptibility across multiple *Drosophila* species tested as preliminary proof of concept. Error bars represent mean ± SEM.
b. Two distinct setups were used to conduct toxicity assays described in the methods. In brief, set up 1 contained DMDS mixed with fly food while set up 2 ensured the presentation of only DMDS vapours through a foam as described earlier [37].
c. A time course representation of *Dmel* regaining consciousness post removal from DMDS-containing substrate. Note that *Dmel* were exposed to DMDS as described earlier for 2 hours. A complete knock-down of 100% of flies followed by transfer to a fresh vial containing normal food was considered t = 0. Fly mobility was scored at hourly intervals. Error bars represent mean ± SEM. n = 6.
d. A time course representation of susceptibility in *Dmel* when presented with multiple concentrations of either DMDS or NaN_3
e. A dose-dependent anaesthesia induction between *Dmel* and *Dbus* using sevoflurane, a known anaesthetic [37]. n = 5.
f. A time course representation of DMDS-induced susceptibility across multiple *Drosophila* species to test the amino acid hypothesis explained in fig 3e.
g. Oviposition preference in *D. mojavensis mojavensis* when presented with a choice between DMDS and mineral oil. Note that no eggs were deposited on either substrate even though flies were mature and mated (>10 days old). Transfer of these flies to normal food vials resulted in the observation of multiple eggs within 24 hrs. n = 12.
h. Representation of multiple *Drosophila* species based on their geographical origin. Color codes denote their classification based on DMDS susceptibility observed in the present study (fig. 3e). Source data are provided as a source data file.

Supplementary tables:

Supplementary table 1: List of odorants used for single sensillum recording experiments. Abbreviations used: Sigma-Aldrich (Steinheim, Germany): SA, Acros Organics B.V.B.A.: AO, Fluka: F, Alfa Aesar: A.A, ABCR GmbH: ABCR, TCI chemicals: TCI. ROTH: RO and Institute stock: Stock

	Odorant	CAS no.	Supplier: Catalogue number
1	Hexane	110-54-3	TCI: S0279
2	Ethyl acetate	141-78-6	SA: 270989
3	Ethyl lactate	97-64-3	SA: W244015
4	CO_2	Mouth aspiration	
5	Methyl salicylate	119-36-8	SA: M6752
6	Methyl acetate	79-20-9	F: 45999
7	Ethyl-3-hydroxybutyrate	5405-41-4	AO: 118540250
8	ethyl hexanoate	123-66-0	SA: 148962
9	2-heptanone	110-43-0	SA: W254401
10	E2-hexanal	6728-26-3	SA: 132659
11	geosmin	16423-19-1	SA: UC18
12	geranyl acetate	105-87-3	Stock (originally SA: 173495)
13	pentyl acetate	628-63-7	SA: W504009
14	1-octen-3-ol	3391-86-4	SA: W280518
15	guaiacol	90-05-1	SA: W253200
16	ethyl benzoate	93-89-0	SA: E12907
17	Ethyl crotonate	623-70-1	SA: 16794-0
18	acetoin	513-86-0	SA: W200808

19	linalool	126-91-0	SA: 74856
20	2 phenyalcohol	60-12-8	SA: 77861
21	benzyl butyrate	103-37-7	Stock (originally A.A: B24241)
22	2-butanone	78-93-3	SA: W217018
23	ethyl butanoate	105-54-4	SA: E15701
24	isopropyl benzoate	939-48-0	Stock (originally ABCR: AB137185)
25	Dimethyl disulphide	624-92-0	SA: 528013
26	acetone	67-64-1	RO: 5025.5
27	methyl benzoate	93-58-3	SA: 18344
28	6-methyl-5-helpten-2-one	110-93-0	SA: W270733
29	Hexyl acetate	142-92-7	SA: 10815-4
30	Isopentyl propionate	105-68-0	SA: W208205
31	2-nonanol	628-99-9	SA: N30307
32	Isopentyl alcohol	123-51-3	SA: w205710
33	1-hexanol	111-27-3	F: 471402
34	2-methyl phenol	95-48-7	F: 60990
35	2-nonanone	821-55-6	SA: N30307
36	Isopentyl acetate	123-92-2	SA: 30696-7
37	4-methylphenol	106-44-5	SA: 61030
38	Acetophenone	98-86-2	Stock (originally SA: 42163)
39	methyl hexanoate	106-70-7	SA: W270806
40	propyl acetate	109-60-4	SA: 133108
41	citral	5392-40-5	SA: C83007
42	2,3-Butanediol	513-85-9	SA: B84904
43	nonanal	124-19-6	SA: W278220

44	phenol	108-95-2	Riedel-de Haën: 33517
A	Dimethylsulfide (DMS)	75-18-3	SA: 528021
B	Dimethyltrisulfide (DMTS)	3658-80-8	SA: W327506
C	Dipropyldisulfide (DPDS)	629-19-6	SA

Supplementary table 2: Diagnostic odors for identified *D. busckii* sensillum classes. Diagnostic odors were used to differentiate between sensillum types and eventually compare them to the known sensillum types described in *D. melanogaster*[14].

D. busckii Sensillum class	Neuron	*D. busckii*
B ab1	ab1A	CO_2
	ab1B	Ethyl lactate
	ab1C	Ethyl acetate
	ab1D	Methyl salicylate
B ab2	ab2A	acetone
	ab2B	Dimethyl disulphide
B ab3	ab3A	Hexyl acetate
	ab3B	3 ITC
B ab4	ab4A	nonanal
	ab4B	Geosmin

B ab5	ab5A	geranyl acetate
	ab5B	oraphan
B ab6	ab6A	1-octen-3-ol
	ab6B	Guaiacol
B ab7	ab7A	isopropyl benzoate
	ab7B	2-nonanone
B ab8	ab8A	Acetoin
	ab8B	Acetone
B ab9	ab9A	geranyl acetate
	ab9B	2-phenylalcohol, Acetophenone
B ab10	ab10A	2-methyl phenol
	ab10B	oraphan
B ab11	ab11A	Hexyl acetate
	ab11B	2-nonanol

Supplementary table 3: A list of all *Drosophila* species used in the study. The species were maintained in the laboratory for several generations. However, these species came originally from either Kyoto stock center (KC) or from the National Drosophila Species Stock Center at Cornell University (CU)

Species	food	stock number/ source
Drosophila ananassae	normal food	14024-0371.11
Drosophila erecta	normal food	14021-0224.01
Drosophila mauritiana	normal food	
Drosophila melanogaster Canton S	normal food	
Drosophila simulans	normal food	
Drosophila suzukii	normal food	14023-0311.00
Drosophila willistoni	normal food	14030-0811.24
Drosophila busckii	Normal: Banana: wheat food (2:0.5:0.2 ratio)	13000-0081.00
Drosophila mojavensis	normal food	15081-1352.10
Drosophila navojoa	normal food	15081-1374.12
Drosophila virilis	normal food	15010-1051.00
Drosophila bipectinata	normal food	14024-0381.00
Drosophila hydei	normal food	15085-1641.03
Drosophila mercatorum	normal food	15082-1521.00
Drosophila immigrans	normal food	15111-1731.00
Drosophila putrida	normal food	15150-2101.00
Drosophila pseudoobscura	normal food	14011-0121.00
Drosophila americana	normal food	15010-0951.00

D. mojavensis baja	normal food	15081-1351.04
Drosophila mettleri	normal food	15081-1502.11
Drosophila ezoana	normal food	E-15701
Drosophila novamexicana	normal food	15010-1031.08

Supplementary table 4: A detailed composition of food types used in this study.

**Normal food components**	unit	500 ml
Treacle	g	59
Brewer's yeast	g	5.4
Agar	g	2.1
Polenta	g	47
Propionic acid	ml	1.2
Nipagin 30%	ml	1.65

**Banana food components**	unit	
Agar	g	85
Yeast	g	165
Methyl paraben	g	13.4
Blended bananas	g	825
Karo syrup	g	570
Liquid malt extract	g	180
100% ethanol	ml	134
Water	L	6

**Wheat food components**	unit	
Semolina (corn based)	g	50
Wheatgerm	g	50
Sugar	g	50
Dry yeast	g	40
Agarose	g	8
Propionic acid	ml	5
Methyl paraben	ml	3.3
Water	L	1
