# Supplementary material for: Preference for and resistance to a toxic sulfur volatile opens up a unique niche in Drosophila busckii
Source: Nat Commun. 2025 Jan 17;16:767. doi: 10.1038/s41467-025-55971-2 (PMC11742422; doi:10.1038/s41467-025-55971-2)
Supplement: Supplementary file 2 — Reporting Summary [file 41467_2025_55971_MOESM2_ESM.pdf]

nature portfolio

Corresponding author(s): Bill S Hansson

Last updated by author(s): 08/11/2024

Reporting Summary

Nature Portfolio wishes to improve the reproducibility of the work that we publish. This form provides structure for consistency and transparency in reporting. For further information on Nature Portfolio policies, see our Editorial Policies and the Editorial Policy Checklist.

Statistics

For all statistical analyses, confirm that the following items are present in the figure legend, table legend, main text, or Methods section.

n/a	Confirmed	
☐	☒	The exact sample size (*n*) for each experimental group/condition, given as a discrete number and unit of measurement
☐	☒	A statement on whether measurements were taken from distinct samples or whether the same sample was measured repeatedly
☐	☒	The statistical test(s) used AND whether they are one- or two-sided<br>*Only common tests should be described solely by name; describe more complex techniques in the Methods section.*
☒	☐	A description of all covariates tested
☐	☒	A description of any assumptions or corrections, such as tests of normality and adjustment for multiple comparisons
☐	☒	A full description of the statistical parameters including central tendency (e.g. means) or other basic estimates (e.g. regression coefficient) AND variation (e.g. standard deviation) or associated estimates of uncertainty (e.g. confidence intervals)
☐	☒	For null hypothesis testing, the test statistic (e.g. *F*, *t*, *r*) with confidence intervals, effect sizes, degrees of freedom and *P* value noted<br>*Give P values as exact values whenever suitable.*
☒	☐	For Bayesian analysis, information on the choice of priors and Markov chain Monte Carlo settings
☒	☐	For hierarchical and complex designs, identification of the appropriate level for tests and full reporting of outcomes
☒	☐	Estimates of effect sizes (e.g. Cohen's *d*, Pearson's *r*), indicating how they were calculated

Our web collection on statistics for biologists contains articles on many of the points above.

Software and code

Policy information about availability of computer code

Data collection	Behavioral data was collected by visual observations. chemical data was collected by GC-MS while electrophysiology data was collected using Syntech software (AutoSpike).
Data analysis	Statistical analysis was performed using Graph pad Prism while electrophysiology data was analyzed using AutoSpike software

For manuscripts utilizing custom algorithms or software that are central to the research but not yet described in published literature, software must be made available to editors and reviewers. We strongly encourage code deposition in a community repository (e.g. GitHub). See the Nature Portfolio guidelines for submitting code & software for further information.

Data

Policy information about availability of data

All manuscripts must include a data availability statement. This statement should provide the following information, where applicable:

- Accession codes, unique identifiers, or web links for publicly available datasets
- A description of any restrictions on data availability
- For clinical datasets or third party data, please ensure that the statement adheres to our policy

All data are available in the main text or in the supplementary materials. Source data are provided as a source data file. For any other data, correspondence and requests should be directed to B.S.H

Research involving human participants, their data, or biological material

Policy information about studies with human participants or human data. See also policy information about sex, gender (identity/presentation), and sexual orientation and race, ethnicity and racism.

Reporting on sex and gender	Not applicable
Reporting on race, ethnicity, or other socially relevant groupings	Not applicable
Population characteristics	Not applicable
Recruitment	Not applicable
Ethics oversight	Not applicable

Note that full information on the approval of the study protocol must also be provided in the manuscript.

Field-specific reporting

Please select the one below that is the best fit for your research. If you are not sure, read the appropriate sections before making your selection.

☒ Life sciences ☐ Behavioural & social sciences ☐ Ecological, evolutionary & environmental sciences

For a reference copy of the document with all sections, see nature.com/documents/nr-reporting-summary-flat.pdf

Life sciences study design

All studies must disclose on these points even when the disclosure is negative.

Sample size	Sample sizes were chosen to at least match or rather exceed those of similar publications. For behavioral assays sample sizes at least matched or exceeded similar previous publications involving studies with drosophilids (Dweck et al., 2013). For electrophysiology, a sample size of 5-7 replicates per stimulus was achieved in case of experiments involving D. busckii. Exception include screening of D. busckii antenna experiments (extended figure 3a) where some sensillum types were highly rare and a low replication number can be observed. In case of experiments involving D. melanogaster antenna screening, a standard replicate number (n=3) was maintained due to the wide availability of literature concerning this model species.
Data exclusions	No data was excluded from the analysis
Replication	All numbers of replications are provided as sample sizes in the different figures as well as individual data points are shown wherever possible. For behavioral and survival experiments, at least two biological replicates were tested and the data was pooled together.
Randomization	For behavioral experiments involving rotting vegetables, stimuli were prepared by pooling multiple samples while at least 2 biological replicates were ensured. For electrophysiology, randomization of stimuli order was performed whenever possible. For survival experiments, involving testing of multiple drosophilid species, any given stimulus was presented to a randomized order of species in order to nullify any temporal effects. Finally, all flies tested in these experiments were collected from different vials and tested across generations to ensure sufficient randomization.
Blinding	Flies from different vials were collected, pooled together and sorted one day before testing.

Reporting for specific materials, systems and methods

We require information from authors about some types of materials, experimental systems and methods used in many studies. Here, indicate whether each material, system or method listed is relevant to your study. If you are not sure if a list item applies to your research, read the appropriate section before selecting a response.

Materials & experimental systems

n/a	Involved in the study
☒	☐ Antibodies
☒	☐ Eukaryotic cell lines
☒	☐ Palaeontology and archaeology
☐	☒ Animals and other organisms
☒	☐ Clinical data
☒	☐ Dual use research of concern
☒	☐ Plants

Methods

n/a	Involved in the study
☒	☐ ChIP-seq
☒	☐ Flow cytometry
☒	☐ MRI-based neuroimaging

Animals and other research organisms

Policy information about studies involving animals; ARRIVE guidelines recommended for reporting animal research, and Sex and Gender in Research

Laboratory animals	A list of all drosophilid species used in this study is provided in supplementary table 3. Drosophila ananassae (14024-0371.11), Drosophila erecta (14021-0224.01), Drosophila mauritiana, Drosophila melanogaster Canton S, Drosophila simulans, Drosophila suzukii (14023-0311.00), Drosophila willistoni (14030-0811.24), Drosophila busckii (13000-0081.00), Drosophila mojavensis (15081-1352.10), Drosophila navojoa (15081 1374.12), Drosophila virilis (15010-1051.00), Drosophila bipectinata (14024-0381.00) Drosophila hydei (15085-1641.03), Drosophila merkatorum (15082-1521.00), Drosophila immigrans (15111-1731.00), Drosophila putrida (15150-2101.00), Drosophila pseudoobscura (14011-0121.00), Drosophila americana (15010-0951.00), D. mojavensis baja (15081-1351.04), Drosophila mettleri (15081-1502.11), Drosophila ezoana (E-15701), Drosophila novamexicana (15010-1031.08),
Wild animals	No wild animals were tested
Reporting on sex	Generally a mix of male and females was used in an approximately 1:1 ratio unless specified otherwise.
Field-collected samples	No samples were collected from the field except rotting potatoes. These were obtained from a local supermarket and a private home.
Ethics oversight	All experiments were conducted with drosophilids and do not require ethical clearance.

Note that full information on the approval of the study protocol must also be provided in the manuscript.

Plants

Seed stocks	Not applicable
Novel plant genotypes	Not applicable
Authentication	Not applicable
